# Molecular Characterization and Global Expression Analysis of Lectin Receptor Kinases in Bread Wheat (*Triticum aestivum*)

**DOI:** 10.1371/journal.pone.0153925

**Published:** 2016-04-25

**Authors:** Shumayla, Shailesh Sharma, Ajay K. Pandey, Kashmir Singh, Santosh Kumar Upadhyay

**Affiliations:** 1 Deparment of Botany, Panjab University, Chandigarh, India, 160014; 2 National Agri-Food Biotechnology Institute, (Department of Biotechnology, Government of India), C-127, Industrial Area, S.A.S. Nagar, Phase 8, Mohali, Punjab, India, 160071l; 3 Deparment of Biotechnology, Panjab University, Chandigarh, India, 160014; National Institute of Plant Genome Research, INDIA

## Abstract

Lectin receptor kinases (LRKs) play a critical role in plants during development and stress conditions, but a comprehensive analysis at genome level is still not carried out in *Triticum aestivum*. Herein, we performed the genome wide identification, characterization and expression analysis of these genes in *T*. *aestivum* (*TaLRK*). In-total 263 *TaLRK* genes were identified, which were further classified into three groups based on the nature of lectin domain. We identified, two *TaLRKs* consisted of calcium-dependent lectin (*C-LRK*), while 84 legume-lectin (*L-LRK*) and 177 bulb-lectin (*B-LRK*) domains. The *L-LRK* and *B-LRK* genes were distributed throughout the genome of *T*. *aestivum*. Most of the *TaLRKs* were clustered as homologs, which were distributed either in proximity on same chromosome or on homoeologous chromosomes of A, B and D sub-genomes. A total of 9 and 58 duplication events were also predicted in *L-LRK* and *B-LRK*, respectively. Phylogenetic analysis indicated conserved evolutionary relationship of homologous and orthologous genes from multiple plant species. Gene ontology analysis indicated TaLRKs role in binding, signaling and receptor activities. Most of the TaLRKs consisted of a trans-membrane domain and predicted to be localized in the plasma-membrane. A diverse expression pattern of *TaLRK* genes was found in various developmental stages and stress conditions. Some *TaLRKs* were found to be highly affected during a particular stress, which indicated a specialized role of each *LRK* gene in a specific stress condition. These results described various characteristic feature and expression pattern of *TaLRK* genes, which will pave the way for functional characterization in wheat.

## Introduction

Plants countenance several challenges like pathogens attack, drought, heat and various other stresses during their life cycle. To protect themselves, plants have evolved strategies for perception, signaling and immune responses [1]. The receptor-like kinases (RLKs) play a crucial role in stimulation of immune responses in plants. They consist of an extracellular domain to receive signal, a trans-membrane domain for anchoring of protein into the membrane and a cytoplasmic serine/threonine kinase domain for immunity stimulation in plants [2–4]. Several RLKs are identified from various plant species, which are involved in development and various biotic and abiotic stress related responses [4].

The lectin receptor kinases (LRKs) are a class of RLKs, which consists of an extracellular lectin domain at N-terminus. On the basis of nature of lectin domain, LRKs are classified into three groups- L-LRK, G-LRK and C-LRK, [5–6]. The L-LRK and C-LRK consists of legume lectin and calcium dependent lectin domain, respectively [7–8]. The G-LRK consists of bulb-lectin (B-lectin) or D-mannose binding lectin domain. The B-lectin was later renamed as GNA-related lectin (G-lectin) [9]; therefore this domain containing LRK was named as G-LRK. Recently, Jiang et al. [10] proposed a new nomenclature of lectins and renamed the GNA related lectin to B-Lectin. Here, the nomenclature of Jiang et al. [10] was followed throughout the study and therefore B-LRK was used in place of G-LRK. The B-LRK is also known as S-domain RLKs, due to the presence of S-locus glycoprotein domain and their role in self-incompatibility in plants [11–12]. Plasminogen-apple-nematode (PAN) and cysteine-rich epidermal growth factor (EGF) like domains are also reported in certain B-LRK [13].

The LRKs play diverse role in plant development and stress management [14–16]. It is reported to be involved in cotton fiber development [17], pollen development [18], symbiotic association of plants [19–21], defense related activities against fungus [22], bacteria [23] and insect pests [24–25], and several other stress responses [5–6, 26–28]. They are characterized from numerous plant species [5–6, 16, 19, 24–25, 27]. However, genome wide identification, characterization and expression analysis of *LRK* genes has not been performed in bread wheat (*T*. *aestivum*), one of the important crop plant of the world. The availability of genome information [29] and several tissue and developmental stage specific transcriptome data [30–31] in the last few years, unfolded the opportunity for the detailed characterization of various gene families in *T*. *aestivum*.

Herein, the available genome and transcriptome information of *T*. *aestivum* was explored to identify all the possible *TaLRK* genes. The identified genes were characterized for genome and chromosome wise categorization, annotation, gene ontology mapping, homologous and orthologous gene identification. The duplication events and phylogenetic relationship were also analyzed. The TaLRK protein sequences were characterized for sequence similarity, domain organization, sub-cellular localization, molecular weight and several other features. Expression analysis of *TaLRK* genes was performed during various developmental stages and stress conditions to reveal their role in these circumstances. To the best of our knowledge, this is the first report of genome wide characterization of LRKs in *T*. *aestivum*.

## Materials and Methods

### Isolation and annotation of *Triticum aestivum lectin receptor kinases (TaLRKs)*

The high-confidence (HCS) gene model nucleotide and protein sequences (99,386) of *T*. *aestivum* were downloaded from IWGSC resources [29] (http://www.wheatgenome.org/, http://wheat-urgi.versailles.inra.fr/Seq-Repository/Genes-annotations) and a local sequence database was developed using the NCBI-blast tool. To find all the LRK family genes, blastp search was performed against the local database using all known LRK sequences from Arabidopsis and rice [6]. In addition, a local pfam (protein family) database was developed by downloading the available pfam data (ftp://ftp.sanger.ac.uk/pub/databases/Pfam) [32]. The *T*. *aestivum* gene model sequences were used for blast search against the pfam database at e-value 10^−5^. All the identified sequences by above stated methods were further verified by blast search against NCBI-conserved domain database (http://www.ncbi.nlm.nih.gov/Structure/bwrpsb/bwrpsb.cgi). The sequences having protein kinase domain (PF00069, CDD|249558) along with either legume lectin (PF00139, CDD|249621) or B-lectin (PF01453 CDD|250632), or C-lectin domain (PF00059 CDD|249550), were considered as putative TaLRKs. The respective domains in each sequence was confirmed using Prosite-Scan (http://prosite.expasy.org/scanprosite/) and SMART (http://smart.embl-heidelberg.de/) servers. Annotation of TaLRK sequences was performed by blast search against the NCBI-nr protein, UNIPROT/SWISSPROT and UNIPROT-UNIREF databases at e-value 10^−6^. The gene ontology (GO) mapping was performed using BLAST2GO server [33].

### Homologs and orthologs prediction, and duplication events and phylogenetic analysis

The homologous *TaLRK* sequences were identified by blast search at e-value 10^−10^ with more than 90% identity with each other and reported *T*. *aestivum* unigene sequences at NCBI unigene database (http://www.ncbi.nlm.nih.gov/UniGene). A non-redundant nomenclature of *TaLRKs* was performed as per their homology and clustering, which was further confirmed by phylogenetic analysis. The proposed nomenclature was followed in later part of our study. The orthologous genes in the related plants like rice, *Hordeum* and *Brachypodium* were predicted using best bidirectional blast hit with e value 10^−10^. The *TaLRK* genes were used as query against the local *LRK* sequences database of other plants. The paralogous genes and duplication events were predicted by blast search at e value 10^−10^ with ≥80% sequence similarity. For phylogenetic analysis, full length sequences were aligned, the aligned fragments were extracted and neighbor joining method with 1000 bootstrap replicates was used for the construction of phylogenetic tree using MEGA 6 [34].

### *In-silico* characterization

The *TaLRK* gene sequences were categorized into their respective sub-genome (A, B, D) and chromosomes (1–7) on the basis of gene model sequence identifier. The chromosomal location and number of exons and introns was obtained by blast search against the available *T*. *aestivum* chromosome sequences at http://plants.ensembl.org/Triticum_aestivum/ and https://urgi.versailles.inra.fr/blast/.

The conserved domains and their positions were identified by blast search against NCBI conserved domain database (http://www.ncbi.nlm.nih.gov/Structure/bwrpsb/bwrpsb.cgi) [35]. The domain architecture was analyzed using SMART (http://smart.embl-heidelberg.de/), Scan-Prosite (http://prosite.expasy.org/scanprosite/) and InterProScan (http://www.ebi.ac.uk/Tools/pfa/iprscan/) servers. The NCBI blast server (http://blast.ncbi.nlm.nih.gov/Blast.cgi) was used for similarity analysis of TaLRK sequences with other proteins in the database. The molecular weight and pI was obtained using Expasy MW/pI tool (http://web.expasy.org/compute_pi/). Signal peptide and cellular localization was analyzed using SignalP 4.1 (http://www.cbs.dtu.dk/services/SignalP/) and CELLO v.2.5 (http://cello.life.nctu.edu.tw/) servers respectively [36–37]. The cellular localization was also confirmed by pSORT server (http://wolfpsort.org/) [38]. The ChloroP (http://www.cbs.dtu.dk/services/ChloroP/) was used to reconfirm the chloroplast localization [39]. The TMHMM server v2.0 (http://www.cbs.dtu.dk/services/TMHMM/) was used for the prediction of trans-membrane region. The conserved motif was identified by MEME Suite GLAM2 version 4.10.1 (http://meme-suite.org/tools/meme) [40] and ConSurf blast (http://consurf.tau.ac.il). ClustalW (http://www.ebi.ac.uk/Tools/msa/clustalw2/), MAFFT (http://www.ebi.ac.uk/Tools/services/rest/mafft/) and muscle [41] were used for the multiple sequence alignments.

### Genome wide expression analysis

Genome wide expression analysis of *TaLRK* genes in various organs and developmental stages was performed using high-throughput RNA sequences data (accession number ERP004714) from 5 organs (root, stem, leaf, spike and grain), each with 3 developmental stages in two biological replicates available at https://urgi.versailles.inra.fr/files/RNASeqWheat/ [31]. The reads per kilobase per million mapped reads (RPKM) value was calculated following the method described [42–43]. The reads were mapped to the *TaLRK* sequences with 100% query coverage and sequence similarity. The mapped reads were counted using a local made python script and RPKM value was calculated. The RPKM values were rechecked by mapping the reads using Bowtie2 [44]. The mapped reads were considered for expression analysis using edgeR package of R software (ver. 3.0.1) with adjusted false discovery rate (p <0.01) [45]. The average RPKM value for biological replicates was calculated and heat-map was generated using Hierarchical clustering explorer 3.5 (http://www.cs.umd.edu/hcil/hce/).

The effect of fungus *Puccinia striiformis* f. sp. tritici (Pst) and *Blumeria graminis* f. sp. tritici (Bgt) on the expression of *TaLRK* genes was analyzed using transcriptome data generated by Zhang et al. [46] after 24h post inoculation in triplicates (accession number PRJNA243835). Expression of *TaLRK* genes under drought, heat and their combination was analyzed using transcriptome data generated by Liu et al. [47]. The data were developed after 1h and 6h incubation in two biological replicates (accession number SRP045409).

### RNA isolation, cDNA synthesis and quantitative real time PCR analysis

To validate the organ specific expression of selected genes, total RNA was isolated from root, shoot, leaf and two developmental stages (10 and 20 DAA) of seeds from *T*. *aestivum cv*. Chinese Spring using the RNeasy Plant Mini Kit (Qiagen, USA) following the manufacturer’s protocol. Genomic DNA contamination was removed using DNase from RNase free kit (Ambion, USA) and cDNA was synthesized by using Superscript III First Strand cDNA Synthesis Kit (Invitrogen, USA). The gene specific primers were designed (S1 Table) and quantitative real time (RT) PCR was performed in three biological replicates by employing ABI7500 Fast System (Applied Biosystems, USA) in 10 μl final volume (containing 5 μl 2× SYBR green master mix, 2 pmole each primer and 100 ng cDNA), following the standard protocol. The specificity of gene was analyzed by amplifying the DNA using genomic DNA as template, primers showing single band amplification, were used in real time PCR. The 18S_rRNA (accession number AJ272181.1) gene was used as internal reference gene as described earlier and relative expression of selected genes was calculated [48]. To analyze the effect of heat, drought and combination of both stresses, the leaf samples from *T*. *aestivum cv*. Chinese Spring were collected after 1h and 6h of treatment as described earlier [47]. cDNA was synthesized from total RNA and RT PCR was performed for selected genes (S1 Table) as described above.

## Results and Discussion

### Isolation and genome wide distribution of *Triticum aestivum* lectin receptor kinases (*TaLRKs*)

The extensive blastp search of known LRK sequences, pfam blast and conserved domain blast identified a total of 263 non-redundant LRK sequences in *T*. *aestivum (*TaLRKs). These were further classified into 84 L-LRKs, 177 B-LRKs and two C-LRKs on the basis of presence of an extracellular legume lectin (PF00139, CDD|249621), bulb lectin (PF01453, CDD|250632) and c-lectin (PF00059, CDD|249550) domain along with the protein kinase (PF00069, CDD|249558) domain, respectively (S1 File). A total of 45 L-LRKs, 32 B-LRKs and 1 C-LRK in arabidopsis, and 72 L-LRKs, 100 B-LRKs and 1 C-LRKs in rice are reported in earlier studies [5–6]. Recently, 38 and 22 L-LRKs are identified in *N*. *benthamiana* and tomato, respectively [49]. The above reports indicated a correlation of the number of LRKs with the size of the plant genome. Since the *T*. *aestivum* consists of a huge (~17 Gb) genome with 99386 predicted high confidence gene models [29], it was presumed that the number of genes in different families will be higher than the other model and crop plants.

**Fig 1:**
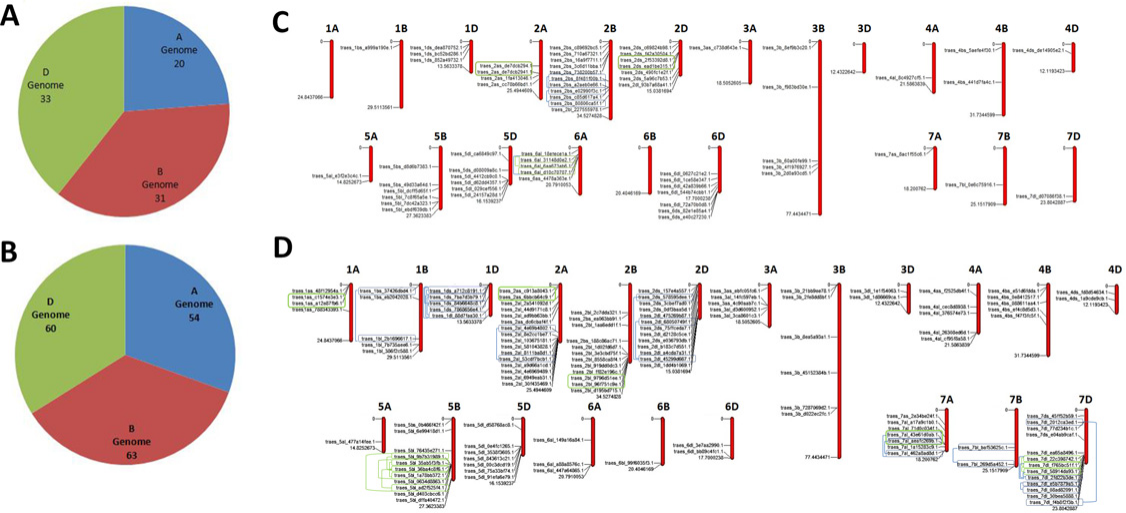
Genome and chromosome wise distribution of *L-LRK* and *B-LRK* genes. Frequency of *L-LRKs* (A) and *B-LRKs* (B) on A, B and D sub-genome of *Triticum aestivum*. Chromosomal distribution of *L-LRKs* (C) and *B-LRKs* (D) genes. The sequences localized over pseudo-molecules (chromosomes) are shown in chromosomal mapping, while those localized over scaffolds could not map. Tandem and segmental duplication events are highlighted and connected by green and blue colour boxes and lines, respectively. The chromosome map was prepared using MapInspect (http://mapinspect.software.informer.com/).

The *TaLRKs* were distributed on each sub-genome (A, B and D) and chromosome of *T*. *aestivum* but at different frequencies (Fig 1, S1 Fig, S2A, S2B and S2C File). A total of 20, 31 and 33 *L-LRKs*; and 54, 63 and 60 *B-LRKs* were detected on A, B and D sub-genomes, respectively (Fig 1A and 1B). However, one *C-LRK* was present on each A and B sub-genome. Out of 84 L-LRKs, 64 were found on pseudo molecules (chromosomes), while others were detected on scaffolds. Similarly, 130 and 47 *B-LRKs* were localized on chromosomes and scaffolds, respectively (S3A, S3B and S3C File). This could be due to the unavailability of complete chromosome sequences of *T*. *aestivum*. The maximum number of *L-LRKs* of A genome was localized on chromosome 6, while in B and D genome on chromosome 2. The *B-LRKs* were mostly localized on chromosome 2 in A and B genome (21 each), while on chromosome 7 in D genome (S1 Fig). Similar distribution of the *LRK* genes is earlier reported in arabidopsis and rice genome [6]. In arabidopsis, most of the *L-LRK* and *B-LRK* genes are localized on chromosome 1 and 3, respectively. However, the highest number of *L-LRKs* is present at chromosome 7 and *B-LRKs* at chromosome 1 in rice [6].

### Prediction of homologs, duplication events and orthologs

The *T*. *aestivum* consisted of ~17 Gb large hexaploid genome with three sub-genomes (A, B and D). This increased the probability of occurrence of homologous sequences, at least thrice (one from each genome) for each gene, unless they are not duplicated or missed during evolution. The homologous sequences across the various sub-genomes were predicted by clustering of isolated *TaLRK* sequences at e-value 10^−10^ with ≥90% similarity with each other. The homology between sequences was also analyzed by their similarity with *T*. *aestivum* unigene clusters, which are the group of known related sequences. The extensive analysis of clustering results indicated the presence of two or more homologous sequences with more than 95% sequence similarity in most of the cases. A total of 76 *L-LRKs*, 172 *B-LRKs* and two *C-LRKs* were clustered into 44, 86 and one distinct clusters, respectively. These closely clustered *TaLRKs* were considered as putative homologs due to their high similarity with each other. The majority of clusters consisted of one or more *TaLRK* sequences from various sub-genomes (S3A, S3B and S3C File). Eight *L-LRKs* and five *B-LRKs* were not clustered with any group. This might be due to the evolvement of new *LRK* genes during evolution by duplication events. The results indicated existence of 44, 86 and one distinct genes for *L-LRK*, *B-LRK* and *C-LRK*, respectively in *T*. *aestivum* with one or more homologs. The number of these distinct genes was quite similar to the other plant species [5–6]. The clustering information was also used for the nomenclature of *TaLRK* genes, one of the distinct sequences was considered as gene while the other clustered sequences as homologs (S3A, S3B and S3C File).

Gene duplication is an important event during the evolution of new genes and development of genetic novelty [50]. It usually occurs by imbalanced crossing over, retro-position and/or genome or chromosome duplication. The allohexaploid genome (AABBDD) of *T*. *aestivum* is known to be developed by hybridization and duplication events, and consisted of more than 80% repeat contents and transposable elements [51–55]. We had also analyzed the contribution of tandem and segmental gene duplications in the genome wide expansion of *L-LRK* and *B-LRK* genes of *T*. *aestivum*. The genes located on the same pseudo-molecule (chromosome) with 80% or more identity at e-value 10^−10^, were considered as duplication events (paralogs). The duplication event within 5 Mb region was considered as tandem duplication (TD), while others as segmental duplication (SD) as reported in case of earlier studies [56]. Moreover, the duplication events localized on scaffolds could not categorize into TD and SD because their actual chromosomal location were not known. A total of nine duplication events were predicted in *L-LRKs*, three on each sub-genome (S4A File, Fig 1C). Three duplication events could categorize as TD, while three as SD. However, three could not categorize due to their localization on scaffolds. In case of *B-LRKs*, 58 duplication events were found. Out of them, 16, 14 and 28 were localized on A, B and D sub-genomes, respectively (S4B File, Fig 1D). An equal number (15) of duplication events were predicted as TD and SD. However 28 duplication events were not categorized. A maximum of 17 duplication events were predicted on chromosome 7D. The results showed duplication events in *B-LRKs* outnumbered the *L-LRKs*, which indicated the major role of duplication events in expansion of *B-LRKs* in *T*. *aestivum*. The expansion of *LRK* gene families by duplication events are also reported in other plants like arabidopsis, rice, *Nicotiana benthamiana*, *Brassica* and soybean [5–6, 49, 57–59]. In such kind of duplication events, it is reported that the original one would retain their function while the newer would perform different role [60–61]. The diverse expression pattern of duplicated genes in the later part of the study supported the above hypothesis in case of *T*. *aestivum* also.

To predict the orthologs of *TaLRKs* in rice, *Hordeum vulgare* (*HV*) and *Brachypodium distachyon* (*Bd*), the best bidirectional blast hit approach was used as reported in earlier studies [56]. The reported *L-LRK* and *B-LRK* sequences [6] of rice were down loaded using gene models identifiers search at http://rice.plantbiology.msu.edu/downloads_gad.shtml. The *LRKs* were earlier not reported in case of *Hv* and *Bd*, therefore their gene model sequences were downloaded from Ensembl plants http://plants.ensembl.org/info/website/ftp/index.html and *LRKs* were predicted by blast search and domain analysis as used in the present study. A total of 62 *L-LRKs*, *161 B-LRKs* and 1 *C-LRK* were found in *Hv*, while 50 *L-LRKs*, 82 *B-LRKs* and 1 C-LRK were predicted in *Bd*. The best bidirectional blast hit indicated 33, 29 and 27 nearby orthologous sequences of *T*. *aestivum L-LRKs* in *Hv*, *Bd* and rice, respectively (S5A File). However, 71, 52 and 40 orthologous *B-LRKs* were predicted in *Hv*, *Bd* and rice, respectively (S5B File). Since the orthologous genes are diverged during speciation event, but they usually maintain the function of their ancestral gene. The results indicated common ancestor of *LRK* genes of *T*. *aestivum*, *Hv*, *Bd* and rice. Though they were separated during speciation, but still maintained their similarity at sequence level, which indicated evolutionary conservation of their function. McClung [62] reported the evolution of monocot and eudicot plants from common ancestor, which further supported our hypothesis.

### Phylogenetic analysis

**Fig 2:**
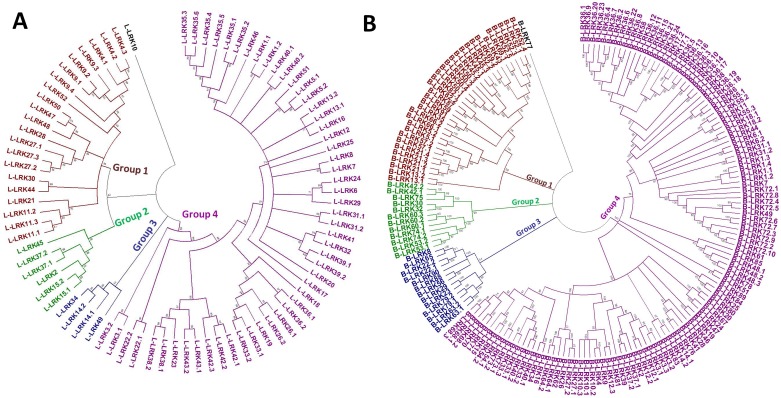
Phylogenetic tree of L-LRKs and B-LRKs constructed by neighbour-joining method with 1000 bootstrap replicates using MEGA 6. Figure shows that both L-LRKs (A) and B-LRKs (B) are classified into four major groups. The homologous sequences are grouped together and follow the proposed nomenclature in present study.

The evolutionary relationship among various groups of TaLRKs was studied by constructing a phylogenetic tree using MEGA 6 [34]. The tree was constructed separately for L-LRKs and B-LRKs using full-length protein sequences (Fig 2A and 2B). The phylogenetic tree showed both L-LRKs and B-LRKs were sub-divided into four groups, and group 4 was largest in both the cases. The various groups were further divided into several subgroups and clades. As per expectation and our proposed nomenclature, the identified homologous LRKs were grouped together with high bootstrap value. It was observed that most of the clades consisted of related LRK sequences, which are probably placed proximally on the chromosome or on the homologous chromosome from A, B and D sub-genomes. To name a few, L-LRK43 with 3 homologs, L-LRK42 and L-LRK38 with 2 homologs, and L-LRK23 grouped together in phylogram, were located on the chromosomes 2AS, 2BS and 2DS in proximity. The phylogenetic relationship of TaLRKs was also analyzed with the LRKs of *Hv*, *Bd* and rice (S2A and S2B Fig). The results showed that the predicted orthologous genes from *Hv*, *Bd* and rice were grouped with their related TaLRK gene in both L-LRKs and B-LRKs phylograms. These phylogenetic analyses indicated evolutionary relationship between each group of TaLRKs and their orthologs from different plants as well. This might be due to the conserveness in various domains and their functions. The phylogenetic relationship between LRK genes and orthologs are also reported in other plants like arabidopsis, rice, *Nicotiana benthamiana* and others [5–6, 49, 59].

### Annotation and gene ontology analysis

**Fig 3:**
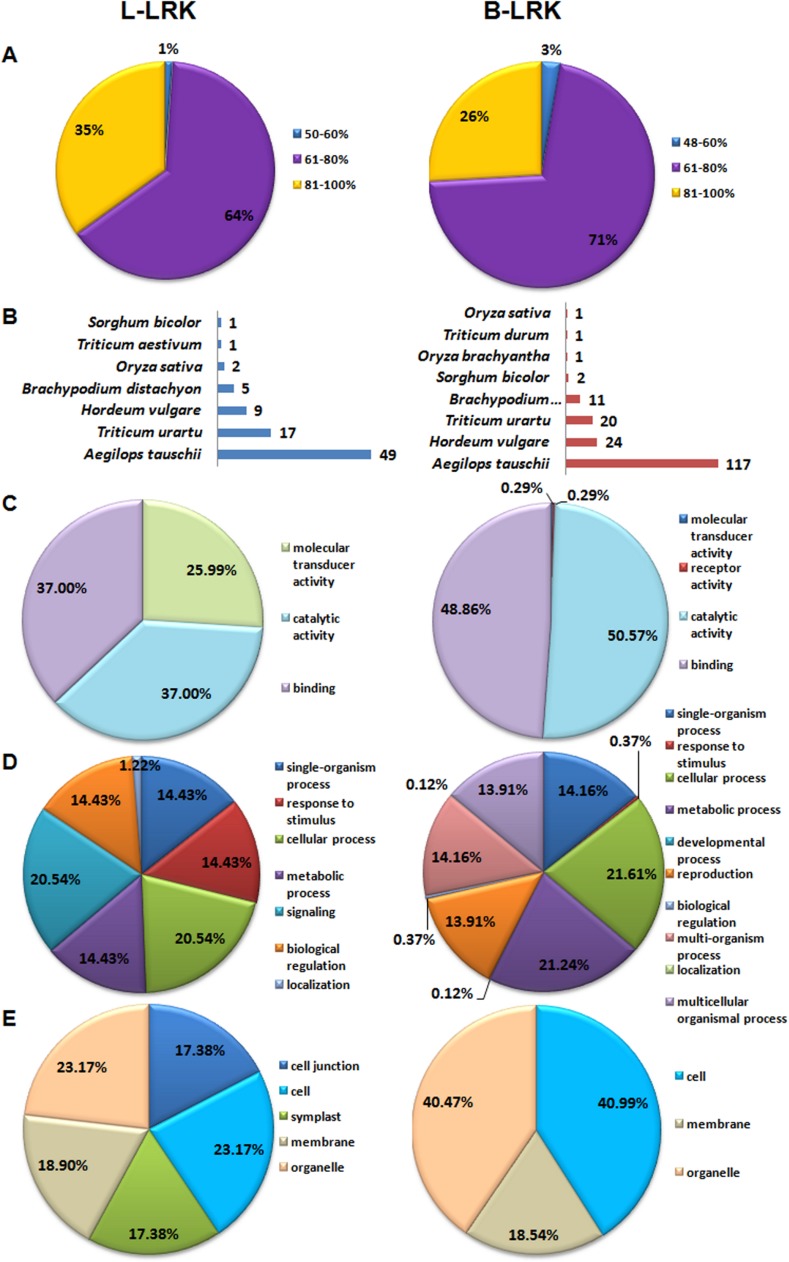
Annotation statistics and gene ontology mapping of *L-LRK* and *B-LRK* genes using Blast2GO tool. (A) Percent similarity and (B) top blast hit species distribution during annotation of *L-LRK* and *B-LRK* genes against NCBI-non redundant protein database. Gene ontology analysis classified *L-LRKs* and *B-LRKs* into (C) molecular function, (D) biological process and (E) cellular component categories.

Annotation of *TaLRK* genes was performed by blast search against various databases (S6A, S6B, S6C, S7A and S7B Files). About 89% *L-LRKs* and 97% *B-LRKs* were annotated with more than 60% similarity at NCBI-nr database (Fig 3A). Most of the *L-LRKs* were annotated as lectin-domain containing receptor kinase, while the *B-LRKs* as serine/threonine-protein kinase receptor. The *C-LRKs* were matched with the predicted proteins. Top blast hit species in annotation were *Aegilops tauschii*, *T*. *urartu* and *H*. *vulgare* (Fig 3B). The blast search against Uniref100 database showed the homology of 84 L-LRKs, 177 B-LRKs and 2 C-LRKs with 66, 128 and 2 distinct Uniref proteins, respectively (S6A, S6B and S6C File).

The gene ontology (GO) analysis using Blast2Go program showed the mapping of 799 GO terms (368 molecular functions, 205 biological processes and 226 cellular components) with L-LRKs and 914 (368 molecular functions, 300 biological processes and 245 cellular components) with B-LRKs (S7A and S7B File). Since more than one GO term was assigned to most of the LRKs, the total number of GO terms could not match to the total number of LRKs. In the molecular function category, catalytic and binding activity was highly represented in both L-LRKs and B-LRKs (Fig 3C). In biological processes, cellular process (21%) and signaling (21%) in L-LRKs, and cellular process (21.6%) and metabolic process (21.2%) in B-LRKs was the most enriched processes (Fig 3D). In cellular components, cell and organelle categories were enriched (Fig 3E). In case of C-LRK, protein phosphorylation was predicted in a biological process, while protein kinase and ATP binding in the molecular function category.

### Gene and protein characterization

The average length of *TaLRKs* coding sequence (CDS) and protein was 1827, 2343 and 1659 bp, and 608, 780 and 552 AA residues for *L-LRKs*, *B-LRKs* and *C-LRKs*, respectively. Most of the *TaLRKs* (62 *L-LRKs* and 95 *B-LRKs*) were intron less. Similar observation is reported in case of other plants [5–6]. A total of 22 *L-LRKs* and 82 *B-LRKs* consisted of one to three, and one to seven introns, respectively. A maximum of 7, 6 and 5 introns were detected in 3, 28 and 11 *B-LRKs*. The *C-LRKs* consisted of 4 exons and 3 introns in each sequence (S3A, S3B and S3C File). A maximum of 3 and 2 introns are reported in arabidopsis and rice *LRK* genes [6], while we found higher number of introns in a few *TaLRK* genes. The number and size of introns is earlier correlated with the size and ploidy level of genomes, however a concrete explanation for the higher number of introns in a few genes are still not available [63–64].

The longest and smallest proteins in each category were L-LRK1.2 and L-LRK36.2, B-LRK29 and B-LRK90, and C-LRK1.1 and C-LRK1.2. The average molecular weight of L-LRKs (~64 kDa) was lower than the B-LRKs (~81 kDa), but higher than C-LRKs (~60 kDa) (Table 1). This could be due to an extra S-locus glycoprotein and/or PAN APPLE domain in some B-LRKs. The isoelectric point (pI) range of L-LRKs, B-LRKs and C-LRKs was 5.35–10.08, 4.94–9.10 and 9.05–9.11, respectively. The average trans-membrane (TM) domain present on L-LRKs (1.42) was higher than B-LRKs (1.12) and lower than C-LRKs (1.5). The L-LRK36.1 consisted of highest 4 TM domains, while 5 L-LRKs and B-LRKs consisted of 3 TM domains. The presence of trans-membrane domain in most of the TaLRKs indicated their membrane binding nature.

**Table 1:** Summary of various characteristic features of TaLRKs.

Characteristics		L-LRK	B-LRK	C-LRK
**Coding sequence length (bp)**	Largest	3579	2931	1665
	Smallest	585	837	1653
	Average	1827	2343	1659
**Nucleotide sequences similarity (%)**	Maximum	100	100	95.88
	Minimum	35	39.38	NA
	Average	62	58.47	NA
**Protein length (AA)**	Largest	1192	976	554
	Smallest	194	278	550
	Average	608	780	552
**Protein sequences similarity (%)**	Maximum	100	100	96.55
	Minimum	10	5.40	0
	Average	40	23.08	0
**Major domains average size (AA)**	Lectin	208	116	125
	Kinase	233	250	262
**Molecular weight (Da)**	Maximum	132613	103056	61243
	Minimum	21379	31755	60686
	Average	64072	81795	60964
**pI**	Maximum	10.08	9.10	9.11
	Minimum	5.35	4.94	9.05
	Average	6.73	6.60	9.08
**Sub cellular localization**	Plasma membrane	69	153	2
	Extracellular	0	20	0
	Nuclear	1	1	0
	Cytoplasmic	5	0	0
	Chloroplast	5	2	0
	Mitochondrial	3	0	0
	Lysosomal	1	1	0
**Signal Peptide**	Present	53	138	2
	Absent	31	39	0
**Transmembrane domains**	Average	1.42	1.12	1.50

Prediction of sub-cellular localization showed that the majority of TaLRKs (69 L-LRKs, 153 B-LRKs and both C-LRKs) were localized in the plasma membrane, while 20 B-LRKs were localized in extracellular region. Others were predicted as nuclear (B-LRK90 and L-LRK3.1), cytoplasmic (5 L-LRKs), chloroplastic (5 L-LRKs and 2 B-LRKs), mitochondrial (3 L-LRKs) and lysosomal (L-LRK51 and B-LRK43). Similar nature of LRKs is also reported in arabidopsis and rice [5–6]. The signal peptide was detected in 53 L-LRKs, 138 B-LRKs and both C-LRKs (Table 1, S3A, S3B and S3C File).

The domain architecture analysis confirmed the presence of respective major domains in each TaLRK proteins (S8A, S8B and S8C File). The kinase domain was present in each gene, however, L-lectin, B-lectin and C-lectin domain was found in L-LRKs, B-LRKs and C-LRKs, respectively. This is the basic characteristic of LRKs [3]. The average length of L-lectin, B-lectin and C-lectin domain was 208, 116 and 125 AA residues, respectively. However, the kinase domain length was 233, 250 and 262 AA residues in L-LRKs, B-LRKs and C-LRKs, respectively. Besides these domains, PAN APPLE and S-locus glycoprotein domains were detected in 169 and 136 B-LRK sequences, respectively. Further, Ephrin type-A receptor 2 transmembrane domain was present in L-LRK52 and B-LRK85 and a DUF3403 in B-LRK46. The diverse nature of LRK gene families indicated independent evolution of each group, which might be due to the different nature of lectin domain in each group. Similar kind of diversity among L-LRKs and B-LRKs is also observed in various other plants [6, 49, 59].

**Fig 4:**
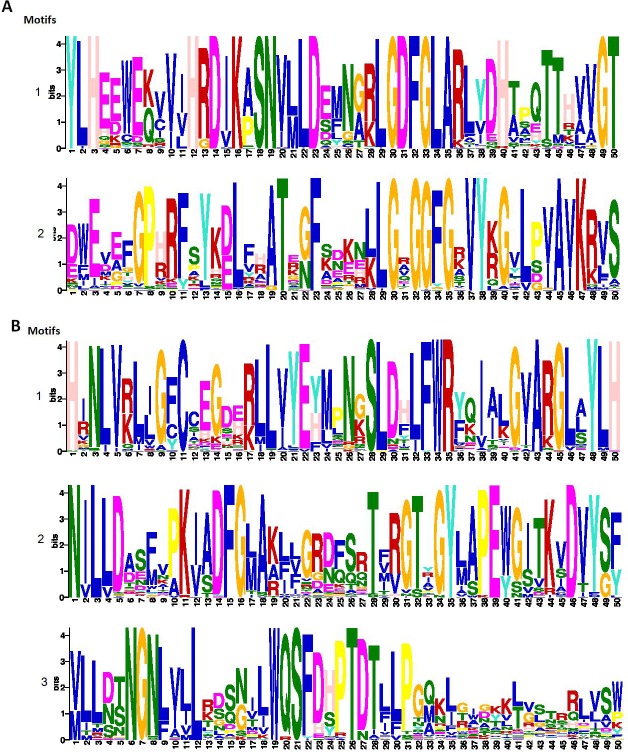
Conserved motifs analysis in L-LRK and B-LRK sequences using MEME suite. Figure (A) shows two highly conserved motifs in L-LRK kinase domain. Figure (B) shows highly conserved motifs of B-LRK kinase (1 and 2) and B-lectin (3) domains.

Besides the above described domains, various other conserved motifs were found in TaLRKs (Fig 4, S3 Fig). A high degree of conservation was observed in kinase domain in comparison to the lectin domain. Conserved motifs were identified in the kinase domain of both the groups (L-LRKs and B-LRKs), which were detected in most of the sequences. The most conserved region in L-LRKs kinase domain was “YLHEEWE[QK][CV]V[IV]HRDIK[AP] SNV[ML]LD [ES][FM]N[AG][KR]LGD FGLARL YDH[AT]PQ TTH[AV]VGT”, detected in 73 out of 84 sequences (Fig 4A). However, the legume lectin domain showed a high diversity (S3A Fig). Similarly in B-LRK, kinase domain motif “H[IR]NLV[KR]L[IL]G[CF]CCEGD[EH[KR] LL[IV]YE[YH] MPN[GK]SLD[FH]LFWR[FY][KQ]I[AI][KL]G[IV]ARGL[ALS]YLH” was found in 170 sequences. The kinase domain of L-LRKs and B-LRKs also consisted of the ATP binding site and activation loop. During signal transduction, the activation loop undergoes conformational change by phosphorylating the Ser, Thr and Tyr residues and activates kinase by allowing binding of substrate to the active site [65]. A conserved motif “[MV]LL[DN][ST]NGN[LF][IV]LL[KR][DS][QS][GN]ILWQSFD[HS]PTDTLLPG[MQ]K [IL] [GT][KY]K[LT][GV][LS] TRRL[L V] SW” was found in 172 sequences in B-lectin domain of B-LRKs (Fig 3B, S3B Fig), which enclosed the reported mannose binding region [66]. The results indicated that besides the diverse nature of lectin domains, which might be associated with the recognition of specific signal by each LRK, certain signature motifs maintained their conservation.

### Expression pattern of *TaLRK* genes

**Fig 5:**
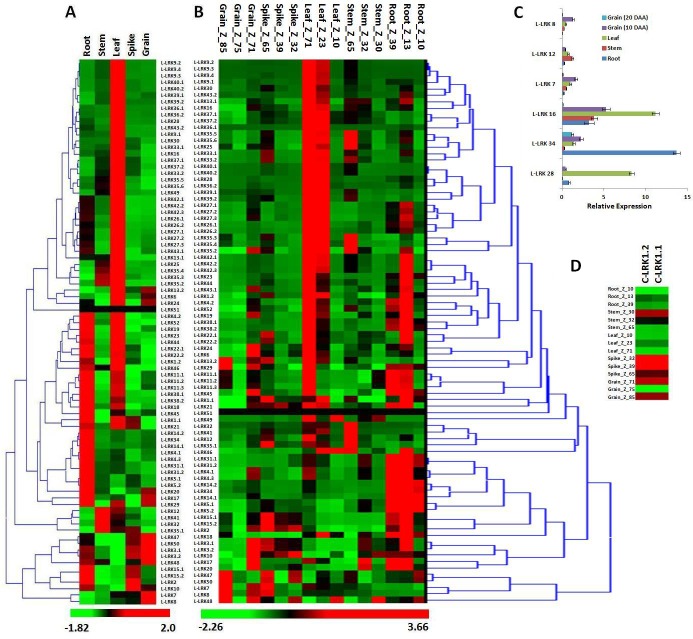
Relative expression profile of *L-LRK* genes in different tissue and developmental stages of *Triticum aestivum*. Heat map shows expression profile of *L-LRK* genes in (A) five different tissues and (B) three developmental stages in each tissue. Quantitative real time PCR analysis of 6 randomly selected *L-LRK* genes (C) shows similar relative expression pattern, as observed with transcriptome data. (D) Heat map shows expression profile of *C-LRK* genes. The developmental stages are denoted in Zadoks scale.

**Fig 6:**
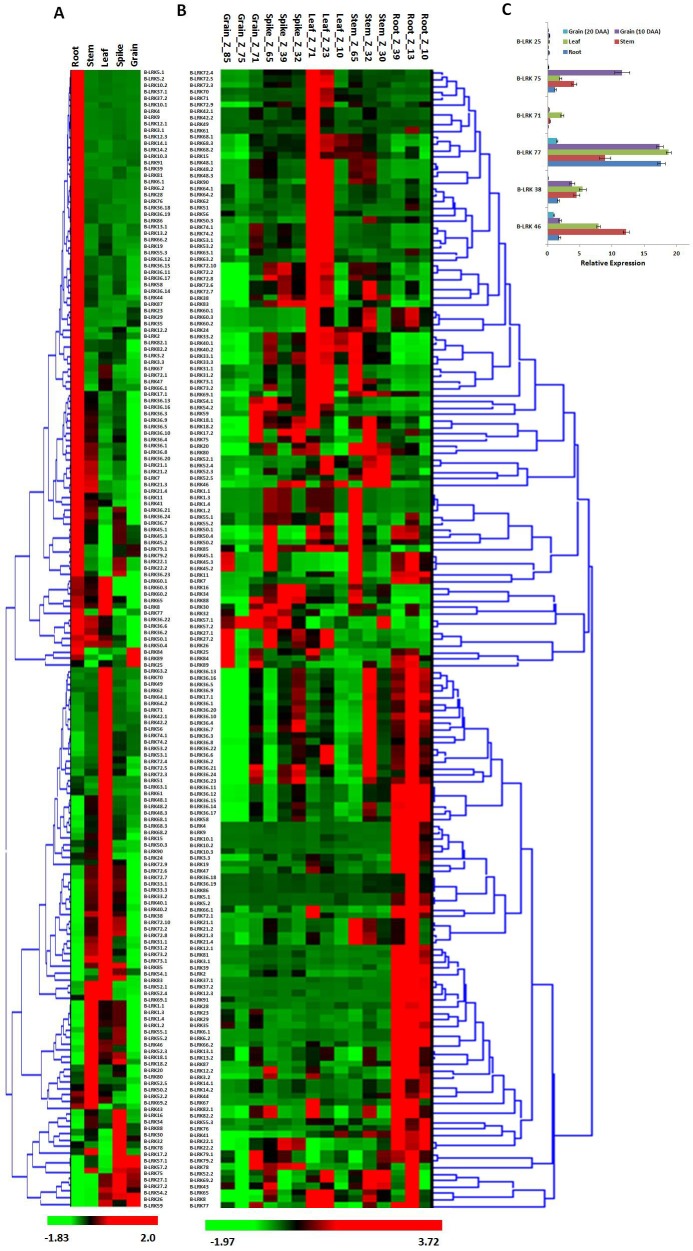
Relative expression profile of *B-LRK* genes in different tissue and developmental stages of *Triticum aestivum*. Heat map shows expression profile of *B-LRK* genes in (A) five different tissues and (B) three developmental stages in each tissue. Validation of six selected *B-LRK* genes (C) by quantitative real time PCR analysis shows comparable relative expression tendency to the transcriptome data. The developmental stages are denoted in Zadoks scale.

We analyzed the relative expression of *TaLRK* genes in 5 different organs (root, shoot, leaf, spike and grain) using the available high-throughput RNA sequences data (accession number ERP004714) [31]. A distinct expression pattern of *L-LRKs* and *B-LRKs* was observed in various organs (Figs 5A and 6A). Most of the genes were exclusively expressed into a specific organ. We observed higher number of *TaLRK* genes expression in leaf and root, as compared to the other organs. About 50% *B-LRKs* and ~42% *L-LRKs* were expressing in high quantity in root as compared to other organs. Similarly, ~60% *L-LRKs* and 28% *B-LRKs* were high expressing in leaf. Only ~2–8% *LRKs* were expressing in the stem, spike and grain (Fig 5A). A few relatively higher expressing genes in each organ were *L-LRK14*.*2* and *L-LRK34* in root, *L-LRK9* and *L-LRK16* in leaf, *L-LRK12* and *L-LRK31* in the shoot, *L-LRK2* and *L-LRK10* in spike, and *L-LRK7* and *L-LRK8* in grain. Similarly, *B-LRK25* and *B-LRK26* in grain, and *B-LRK16* and *B-LRK29* in spike were detected in relatively higher quantity. While several *B-LRKs* were found high expressing in root, stem and leaf similar to the *L-LRK* (Fig 6A). The expression of *C-LRK* was about similar in all the organs (Fig 5D). The organ specific expression of six *L-LRKs* and six *B-LRK*s was further validated using quantitative RT PCR. The results were consistent with the high-throughput RNA sequences data (Figs 5C and 6C).

The expression analysis was also performed in three developmental stages of 5 organs (root, shoot, leaf, spike and grain) as per the Zadoks scale (Figs 5B and 6B). The developmental stage specific expression suggested that most of the genes were expressed at a specific time in each tissue. Most of the *LRKs*, earlier categorized to a specific organ, were actually found as stage specific genes. In case of root, leaf and stem, higher number and quantity of expression of *L-LRKs* and *B-LRKs* was observed in later stages. Intriguingly, we observed higher expression of *L-LRKs* and *B-LRKs* in Z_71 and Z_85 stage as compared to the middle Z_75 stage in grain. In spike, higher numbers of expressing genes were at Z_65 stage (Figs 5B and 6B).

Genome wide distribution of high expressing *TaLRK* genes was also analyzed. Top ten high expressing *L-LRK* and *B-LRK* genes were selected for each developmental stage and analyzed for their genome and chromosome wise distribution (S4A and S4B Fig). A total of 31 *L-LRKs* and 51 *B-LRKs* were found to be highly expressed in one or more developmental stages. These high expressing genes were unevenly distributed on all three sub-genome. The D sub-genome consisted of 13 high expressing *L-LRK* genes, which was higher than B (12 *L-LRKs*) and A (6 *L-LRKs*) sub-genome. However, the B sub-genome consisted a higher number of *B-LRKs* (22 genes) as compared to the A (17 *B-LRKs*) and D (12 *B-LRKs*) sub-genome. The higher numbers of high expressing L-LRKs were found at chromosome 5B and 6D, however *B-LRKs* at chromosome 2A and B.

The above results indicated that the number and expression of genes might be associated with the exposure area of each organ. Since roots and leaves are highly exposed portion of *T*. *aestivum* plants, they showed higher number and expression of *LRK* genes. A few *LRK* genes were specifically expressed in seed developmental stages, which might be related to the development of seed or any specific tissue. But the specific role of each gene in organ, developmental stages or specific tissue needs to be validated individually by using various functional genomics tools in future studies.

### Expression analysis under biotic stress

*Puccinia striiformis* f. sp. *tritici* (Pst) and *Blumeria graminis* f. sp. *tritici* (Bgt) are very common fungal pathogen of bread wheat, which cause stripe rust and powdery mildew diseases, respectively. In order to draw the relation for TaLRKs during the pathogen attack, we studied the effect of Pst and Bgt after 24 h of inoculation using the high-throughput RNA sequences data generated by Zhang et al. [46]. This period was earlier reported as an important time point for wheat to respond fungal attack by expressing early defense related genes [46].

**Fig 7:**
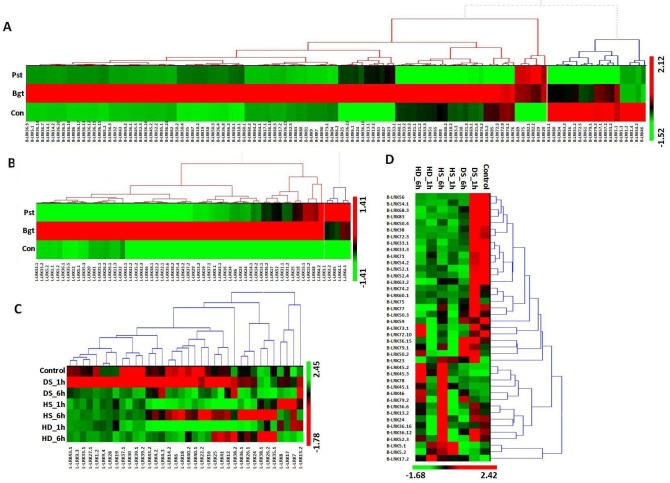
Expression profiling of *L-LRK* and *B-LRK* genes during biotic and abiotic stress. Heat map shows the relative expression profile of ≥2 fold up or down regulated (A) *B-LRKs* and (B) *L-LRKs* during biotic stress, and (C) *L-LRK*s and (D) *B-LRK*s during abiotic stress (Bgt: after *Blumeria graminis* inoculation, Pst: after *Puccinia striiformis* inoculation, HS: heat stress, DS: drought stress, HD: heat plus drought stress, Con: control).

The relative expression analysis of *TaLRKs* showed distinct changes in expression pattern under infected conditions. Only ≥2 folds down or up regulated genes after inoculation were considered as affected genes. Higher number of *TaLRK* genes was affected by Bgt than Pst, which indicated diverse responses and specificity of *TaLRKs* towards various stimuli. A total of 120 *B-LRKs* and 60 *L-LRKs* expressions were affected (Fig 7A and 7B, S9A and S9B File). The expression of *C-LRKs* was unaffected. In Pst inoculation, about 25% affected *L-LRKs* were up regulated while ~10% were down regulated. A few highly up-regulated genes were *L-LRK3*.*2* (6.9 F), *L-LRK3*.*1* (6.8 F) and *L-LRK13*.*2* (6.5 F), while *L-LRK1*.*1* (13 F) was extremely down-regulated. In case of Bgt inoculation, interestingly more than 90% affected *L-LRKs* were up regulated while none was down regulated. The *L-LRK13*.*2* (49 F) and *L-LRK38*.*2* (16 F) were exceptionally up-regulated.

In case of B-LRKs, ~12% affected genes were up regulated while ~30% were down regulated after Pst inoculation. The *B-LRK35* (16 F) was significantly up regulated, while *B-LRK31*.*2* (21 F) and *B-LRK57* (15 F) were highly down regulated. In Bgt inoculation, ~75% *B-LRKs* were up regulated while ~8% were down regulated. The *B-LRK35* (76 F) and *B-LRK31*.*1* (7 F) were highly up and down regulated genes, respectively (Fig 7A and 7B).

Differential expression of *LRK* genes during various kinds of biotic stress was earlier reported in several plants [6, 49, 67]. Furthermore, the transgenic expression of *LRK* genes showed varied tolerance against several pathogens like *Phytophthora brassicae*, *P*. *capsici*, *Alternaria brassicicola*, *Magnaporthe grisea*, *Pseudomonas syringae* [49, 67, 68]. The changes in expression pattern of *TaLRKs* in response to the pathogen Pst and Bgt indicated their association with biotic stress tolerance, which needs to be further validated in subsequent studies.

### Specifically expressed *LRKs* during abiotic stress

**Fig 8:**
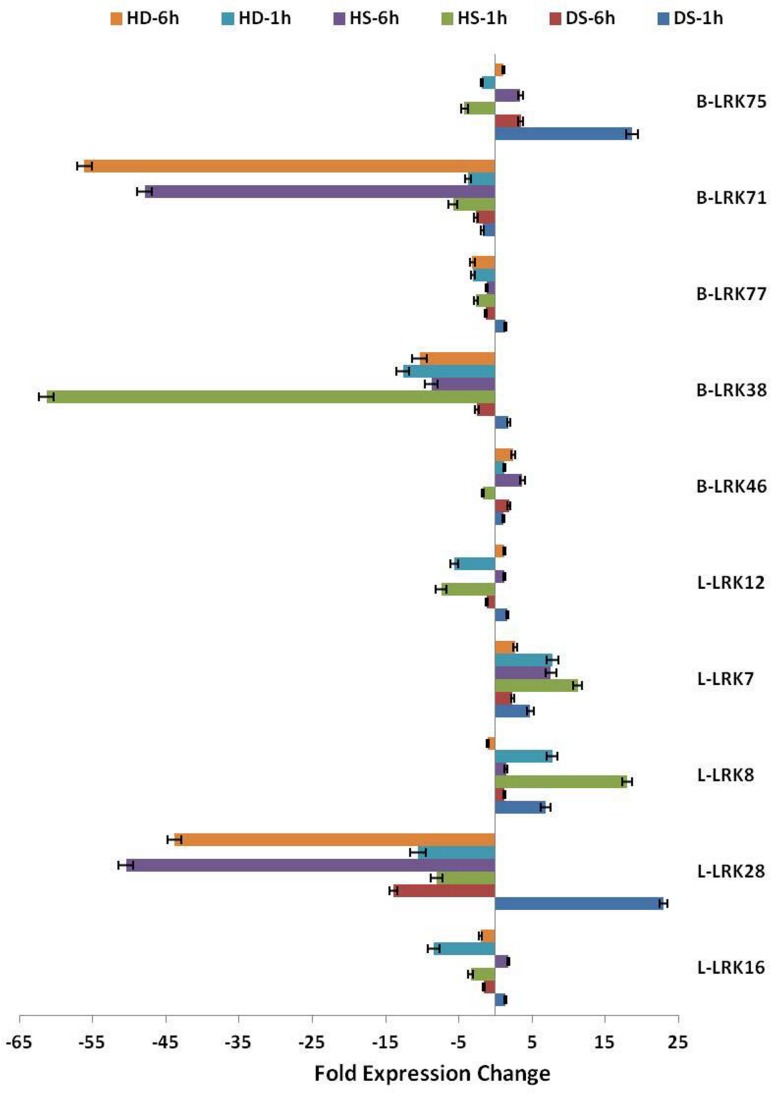
Real time expression analysis of selected *L-LRK* and *B-LRK* genes during abiotic stress. Figure shows fold change in the expression of selected *L-LRK* and *B-LRK* genes under various abiotic stresses as compared to the control condition. The analysis of done with three biological replicates (HS: heat stress, DS: drought stress, HD: heat plus drought stress).

Abiotic stresses like heat, drought and their combination are reported to cause high yield loss in wheat production by affecting the various physiological and biochemical processes [69–70]. It has also been observed that both heat and drought stress act in a synergistic manner in wheat [47]. Therefore, we studied the expression of *TaLRKs* during both these stresses separately and in combination using high-throughput RNA sequences data developed by Liu et al. [47]. The genes showing ≥2 folds down or up-regulation were considered for analysis. A total of 36 *L-LRKs* and 41 *B-LRKs* were affected either by heat (HS) or drought (DS) or in combination of both (HD) (Fig 7C and 7D, S10A and S10B File). A total of 14 *L-LRKs* in DS 1h, three in DS 6h, five in HS 1h, six in HS 6h, three in HD 1h and four in HD 6h were up regulated, in which *L-LRK7* and *L-LRK8* were up regulated during all the stresses. However, 22 *L-LRKs* in DS 6h, 26 in HS 1h, 3 in HS 6h, 27 in HD 1h and 14 in HD 6h were down regulated. The *L-LRK28* was down regulated during all the stresses except DS 1h, where it was 22 folds up regulated (Fig 7C). The exceptionally up regulated *L-LRKs* were *L-LRK28* (22 F) in DS 1h and *L-LRK8* (18 F) in HS 1h, however extremely down regulated were *L-LRK9*.*4* (33 F) in DS 6h and *L-LRK33*.*1* (55 F) in HS 1h, *L-LRK28* (54 F) in HS 6h and *L-LRK37*.*1* (35 F) in HD 1h. The expression of five *L-LRK* genes was validated by quantitative real time PCR. The results were found similar as observed in the transcriptome data (Fig 8).

In case of *B-LRKs*, seven in each DS 1h, DS 6h and HS 1h, 13 in HS 6h, five in HD 1h and eight in HD 6h were up regulated, in which *B-LRK17*.*2* was up regulated during all the stresses. However, two *B-LRKs* in DS 1h, 20 in DS 6h, 29 in HS 1h, 17 in HS 6h, 26 in HD 1h and 19 in HD 6h were down regulated. A total of 15 *B-LRKs* were down regulated during all the stress conditions except DS 1h, where they were unaffected. A few significantly up regulated *B-LRKs* were *B-LRK75* (18 F) in DS 1h, *B-LRK17*.*2* (16 F) in HD 1h, and *B-LRK45* in HS 6h (40 F) and HD 6h (26 F). However, the extremely down regulated *B-LRKs* were *B-LRK52*.*4* (49 F), *B-LRK52*.*1* (24 F) in DS 6h, *B-LRK38* (61 F) in HS 1h, *B-LRK71* (47 F), *B-LRK54* (40 F) and *B-LRK72*.*3* (21 F) in HS 6h, *B-LRK54*.*1* (40 F) in HD 1h, and *B-LRK71* (56 F), *B-LRK33*.*3* (28 F) and *B-LRK72*.*3* (27 F) in HD 6h (Fig 7D). The quantitative real time PCR analysis of few selected *B-LRK* genes showed a similar expression tendency as depicted by the transcriptome data (Fig 8). The expression pattern of *B-LRK* and *L-LRK* genes of rice and arabidopsis is also reported to be affected under various abiotic stresses like drought and heat [6].

## Conclusions

The present study increased our knowledge about the behavior and role of *LRK* genes in one of the important crop plant. We explored the members of LRK families in hexaploid bread wheat, their domain organization, several characteristic features and evolutionary relationship. And most importantly, the expression pattern of these genes was analyzed in various developmental stages and stress conditions. Most of the *L-LRK* and *B-LRK* genes were expressed in relatively higher quantity in leaves and roots. The results revealed stress specific responses of *L-LRK* and *B-LRK* genes. In general our data suggests that the biotic stress affected more genes than the abiotic stress. Interestingly a few genes showed specificity for their regulation during various biotic and/or abiotic stresses. Although, we could not find an association between affected genes under biotic and abiotic stress, but same or related genes were affected under similar stress condition. For example, a few genes like *L-LRK7*, *L-LRK8* and *B-LRK17*.*2* were found up regulated during all the abiotic stress conditions. Further, we found that the genes having very low expression during normal developmental stages were extremely affected in stress conditions. To name a few, the genes *L-LRK13*.*2*, *L-LRK38*.*2*, *B-LRK35* in biotic stress, and *L-LRK7*, *L-LRK8*, *B-LRK52*.*4*, *B-LRK52*.*1*., *B-LRK71* and *B-LRK54*.*1* in abiotic stress were extremely up-regulated in leaves, while their expression was very low under normal condition. This behavior might not be a universal phenomenon as we observed continuous expression or no expression of some genes during all the stages. Moreover, the expression of *L-LRK7* and *L-LRK8* were relatively higher in seed as compared to other tested organs. This could be because of the fact that the seeds are exposed to the elevated temperatures during development. These selected genes can be further characterized for their functional role for the stress management in plants. Our results would pave the way to establish the specific role of each *LRK* gene in different conditions by functional genomics and their encashment by agricultural applications.

## Supporting Information

S1 Fig Genome and chromosome wise distribution of *L-LRK* and *B-LRK* genes. Figure shows distribution of *L-LRK* (A) and *B-LRK* (B) genes on various chromosomes of *Triticum aestivum*.
(TIF)

S2 Fig Phylogenetic analysis of TaLRKs with orthologous genes from rice, *Hordeum vulgare* and *Brachypodium distachyon*. Figure shows phylogenetic relationship of (A) L-LRKs and (B) B-LRKs of *T*. *aestivum* with their orthologous genes from rice, *H*. *vulgare* (HV) and *B*. *distachyon* (Bd). The homologous and nearby orthologous genes are clustered together.
(TIF)

S3 Fig Analysis of conserved sequences using Conserf blast. Figure shows the conserved amino acid sequences, motifs and domains in (A) L-LRK and (B) B-LRK proteins of *Triticum aestivum*. Black, brown and purple underlined regions depict kinase, L-lectin and B-lectin domain, respectively. Grey, pink and red underlined regions are ATP binding site, activation loop and mannose binding sites, respectively.
(TIF)

S4 Fig Genome and chromosome wise distribution analysis of high expressing L-LRK and B-LRK genes. Figure shows distribution of (A) L-LRK and (B) B-LRK on *Triticum aestivum* genome and chromosomes.
(TIF)

S1 File List of gene models categorized as L-LRK, B-LRK and C-LRK. (XLS)

S2 File Distribution of (A) L-type, (B) B-type and (C) C-type Lectin Receptor Kinases on *Triticum aestivum* A, B and D genomes and chromosomes.
(XLS)

S3 File Unigene clustering, nomenclature, chromosomal localization and characterization of (A) L-type, (B) B-type and (C) C-type Lectin Receptor Kinases.
(XLS)

S4 File Genome and chromosome wide duplication events (paralogs) in (A) L-type and (B) B-type Lectin Receptor Kinases.
(XLS)

S5 File Predicted orthologs of (A) L-type and (B) B-type Lectin Receptor Kinases in *Hordeum vulgare*, Rice and *Brachypodium distachyon*.
(XLS)

S6 File Annotation of (A) L-type, (B) B-type and (C) C-type Lectin Receptor Kinases using various database at e-value 10–6.
(XLS)

S7 File Annotation and gene ontology mapping of (A) L-type and (B) B-type Lectin Receptor Kinases using Blast2GO at e-value 10–6.
(XLS)

S8 File Analysis of two major domains Lectin and Kinase in (A) L-LRK, (B) B-LRK and (C) C-LRK.
(XLS)

S9 File Fold expression change in (A) L-type Lectin Receptor Kinases (L-LRK) and B-type Lectin Receptor Kinases (B-LRK) during biotic stress. Two folds or more up regulated and down regulated genes are shown in pink and green colour cells, respectively.
(XLS)

S10 File expression change in (A) L-type Lectin Receptor Kinases (L-LRK) and (B) B-type Lectin Receptor Kinases (B-LRK) during abiotic stress. Two folds or more up regulated and down regulated genes are shown in pink and green colour cells, respectively.
(XLS)

S1 Table List of primers used for quantitative real time PCR. (XLS)
