# Supplementary figures and images for: Molecular Characterization and Global Expression Analysis of Lectin Receptor Kinases in Bread Wheat (Triticum aestivum)
Source: PLoS One. 2016 Apr 25;11(4):e0153925. doi: 10.1371/journal.pone.0153925 (PMC4844157; doi:10.1371/journal.pone.0153925)

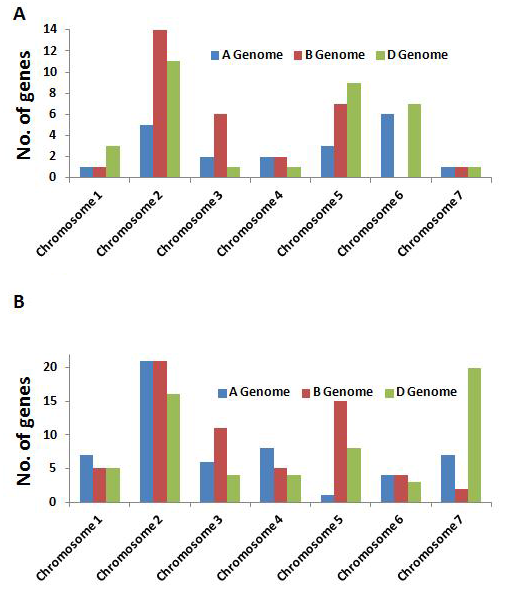

Supplement: S1 Fig — Figure shows distribution of L-LRK (A) and B-LRK (B) genes on various chromosomes of Triticum aestivum. (TIF) [file pone.0153925.s001.tif]

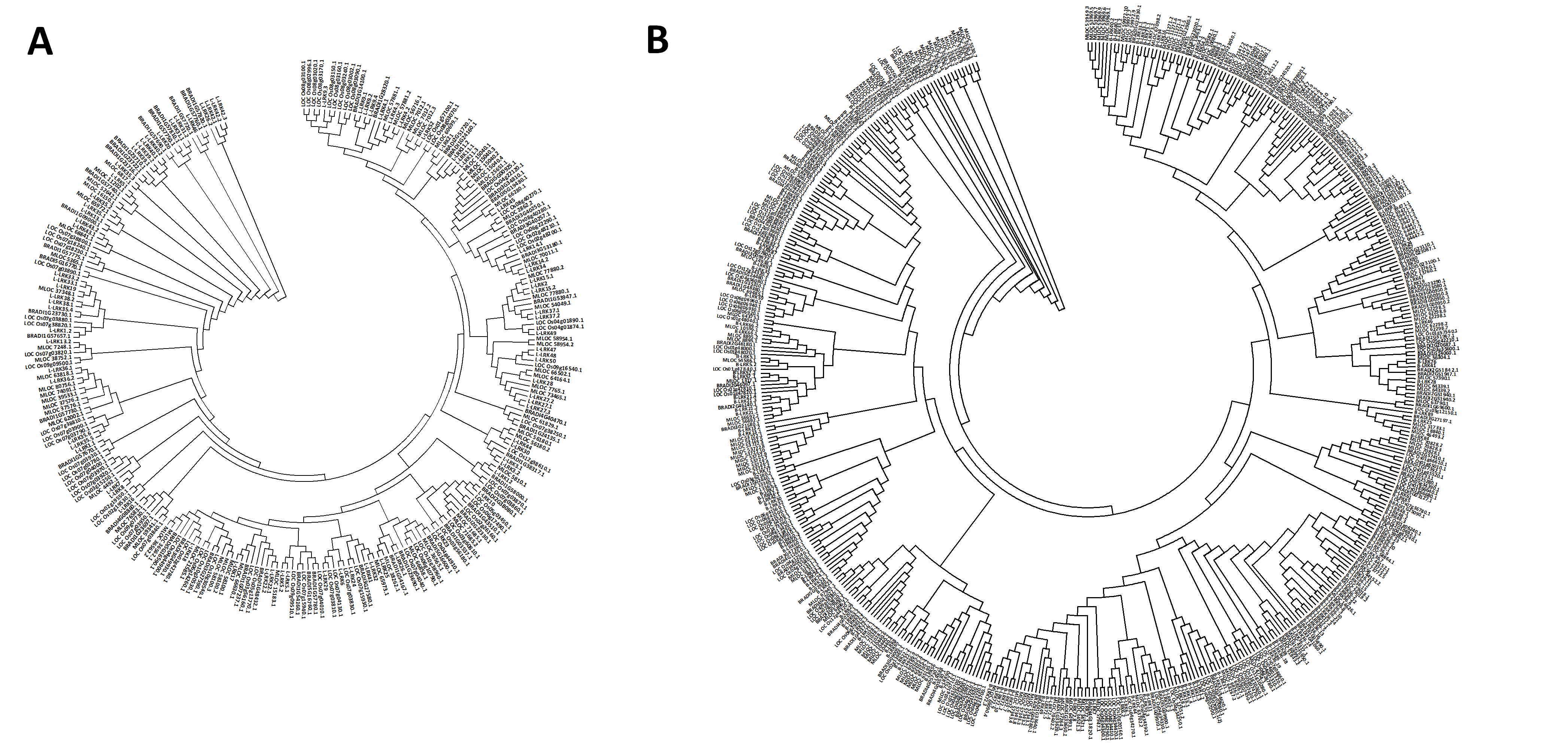

Supplement: S2 Fig — Figure shows phylogenetic relationship of (A) L-LRKs and (B) B-LRKs of T. aestivum with their orthologous genes from rice, H. vulgare (HV) and B. distachyon (Bd). The homologous and nearby orthologous genes are clustered together. (TIF) [file pone.0153925.s002.tif]

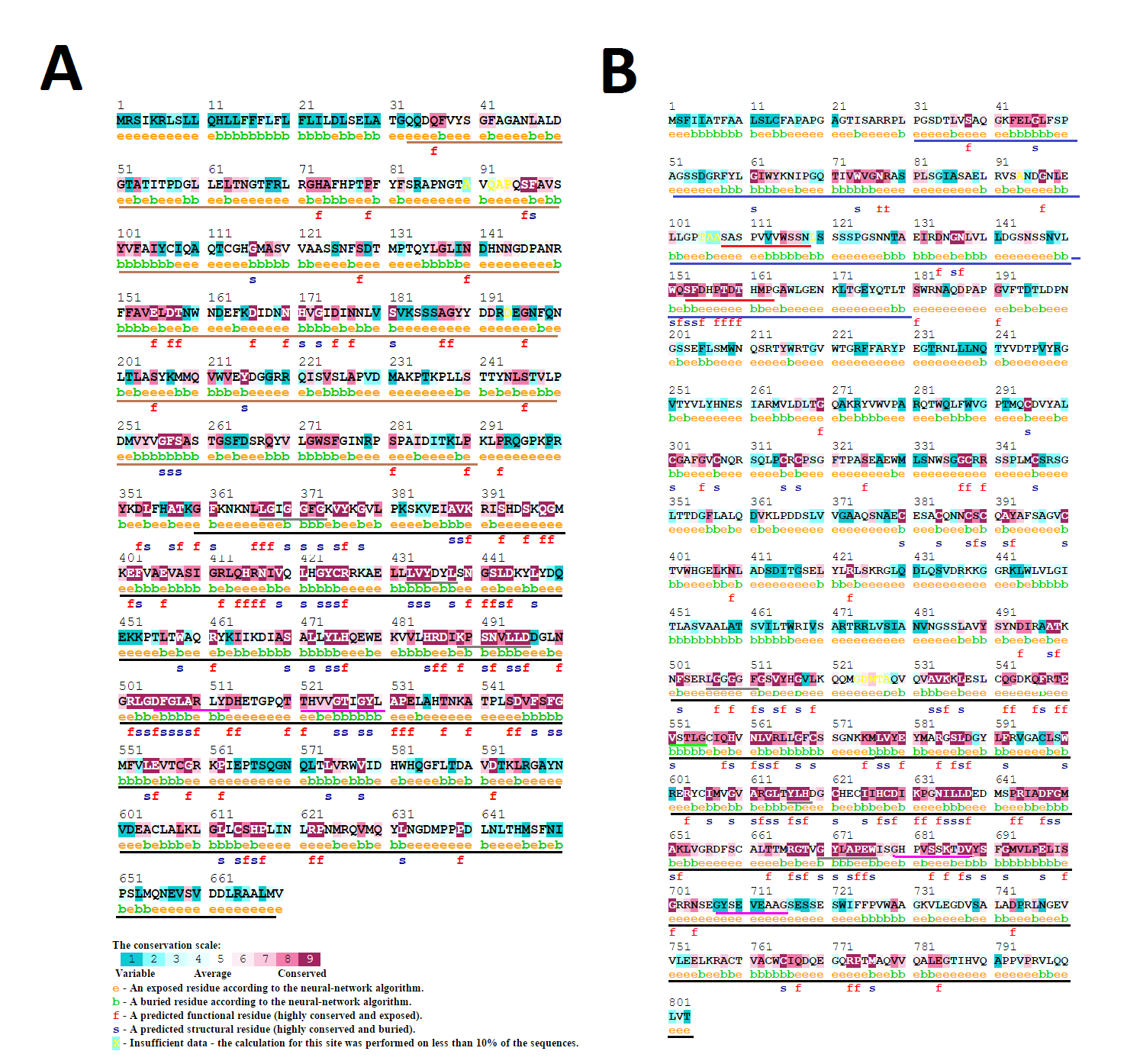

Supplement: S3 Fig — Figure shows the conserved amino acid sequences, motifs and domains in (A) L-LRK and (B) B-LRK proteins of Triticum aestivum. Black, brown and purple underlined regions depict kinase, L-lectin and B-lectin domain, respectively. Grey, pink and red underlined regions are ATP binding site, activation loop and mannose binding sites, respectively. (TIF) [file pone.0153925.s003.tif]

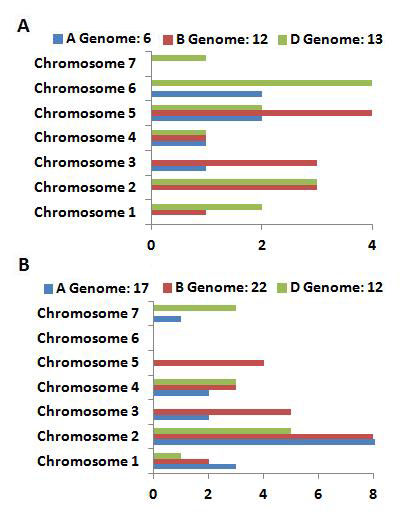

Supplement: S4 Fig — Figure shows distribution of (A) L-LRK and (B) B-LRK on Triticum aestivum genome and chromosomes. (TIF) [file pone.0153925.s004.tif]
